# Immuno-oncology in head and neck squamous cell carcinoma - a narrative review

**DOI:** 10.1590/1414-431X2023e12703

**Published:** 2023-03-17

**Authors:** A.T. da Silva, A.C. Simões e Silva, A. Petroianu

**Affiliations:** 1 Faculdade de Medicina, Universidade Federal de Minas Gerais, Belo Horizonte, MG, Brasil

**Keywords:** Cancer, Head and neck, Squamous cell carcinoma, Immuno-oncology, Immunology, Immunotherapy

## Abstract

Immuno-oncology studies the immune system in cancer. In recent decades, immunotherapy has shown a good response to the treatment of various locally advanced and metastatic cancers. The main mechanisms of action include stimulation of the patient's own immune system to enhance immune responses acting in tumor escape pathways. This review examined the literature related to immune system mechanisms in head and neck squamous cell carcinoma (HNSCC) and their application in immunotherapy using biomarkers. The PUBMED, LILACS, MEDLINE, WHOLIS, and SCIELO databases were searched using the terms squamous cell carcinoma, head and neck, immuno-oncology, immunotherapy, and immunology. The main drugs currently available for clinical use in patients diagnosed with HNSCC include pembrolizumab and nivolumab, both classified as check-point inhibitors. These immunobiological agents improve patient survival and quality of life. Many authors and clinical trials point out that the recommendation of these agents is linked to the dose of PD-L1 (ligand expressed primarily by tumor cells), which proved to be an unreliable biomarker in the patient selection. Recommendation of immunotherapy depends on reliable biomarkers that must be identified in order to achieve good therapeutic results.

## Introduction

Head and neck squamous cell carcinoma (HNSCC) includes a variety of tumors in the oral cavity, nasopharynx, oropharynx, hypopharynx, and larynx that differ in their epidemiology, etiology, and therapeutic approach (1,2). HNSCC is the sixth most common cancer in the world and is responsible for 6% of all cancers and 2% of all cancer-related deaths. The most common triggering factors are the use of tobacco and alcohol, polycyclic aromatic hydrocarbons, nitrosamines, aldehydes, aromatic amines, and human papillomavirus (HPV) subtype 16 (1,2).

Carcinogenetic agents cause epigenetic mutations and alterations in the tumor suppressor oncogenes and genes, triggering uncontrolled cell proliferation and the formation of malignant tumors. HNSCC is heterogeneous from the molecular point of view, characterized by complex alterations of multiple genes. Among the most commonly studied gene alterations are the T-p53 tumor suppressor gene and the gene that codifies the epidermal growth factor receptor (EGFR). These mutations produce proliferative cells that are unmanageable, observed in half of the HNSCC patients (3,4).

The immune response against tumors is primarily mediated by T lymphocytes (5,6). The products generated by mutated tumor suppressor oncogenes and genes are proteins that can be degraded and presented to class I and II MHC molecules in dendritic cells, which lead to phagocytosis of tumor cells (4). Tumor antigens are responsible for inducing immune responses in their host, and some of them constitute potential targets for immunotherapy, being also useful markers for diagnosis and prognosis (5).

The objective of this study was to investigate the literature related to immune system mechanisms in HNSCC and their application in immunotherapy using biomarkers.

This is a narrative review of the literature on immuno-oncology concerning HNSCC based on references found in PUBMED, LILACS, MEDLINE, WHOLIS, and SCIELO databases, using the following key words: squamous cell carcinoma, head and neck, immune-oncology, immunotherapy, and immunology.

## Immune mechanisms in cancer

The challenge in oncology today is to determine which immune response mechanisms can contribute to protection against tumors and to develop means to improve these mechanisms, specifically for each type of tumor (6). The determination of the immune response of cancer patients is necessary to identify mechanisms of action and modulate these processes to obtain good treatment responses in advanced and metastatic cancers. Immunotherapy interferes with the patient's immune system in a way that enhances the immune system's response, acting on the tumor's escape mechanisms. However, current immunotherapy used to treat HNSCC generates major systemic toxicity and is not indicated for local administration (7).

Carcinogenesis caused by HPV, like alcoholism- and smoking-induced carcinogenesis, results from immunodeficiency. Consequently, tumor cells escape recognition and lysis by cytotoxic T lymphocytes and avoid adaptive immunity. This expression is associated with a reduction in the number of lymphocytes caused by the apoptosis of T lymphocytes, decrease in natural killer (NK) cell activity, and decrease in antigen presentation (1).

The balance between T lymphocytes and the tumor microenvironment enables the modulation of anti-tumor immune response (8). Functional alterations of T lymphocytes, macrophages, dendritic cells, and NK cells modify the immune response. Regulatory T lymphocytes and low levels of CD4+ and CD8+ lymphocytes are responsible for immune-modulation in active HNSCC (8). These tumors cause modifications in immune system cells in the tumor microenvironment (9). Once recognized as antigens, pre-malignant cells are destroyed by the immune system, preventing tumor appearance. In contrast, suppressor T cells promote tumor progression (9).

## Strategies for immunotherapy and monoclonal antibodies

There are two main strategies for immunotherapy, with the specific antigen and with the non-specific antigen, both limited by specific mutations of each tumor. The non-specific antigens stimulate anti-tumor mediators and block the immunosuppression induced by the tumor. On the other hand, the specific antigen induces the immune response that is localized and directed only toward tumor cells. In HNSCC, the check-point inhibitor mechanisms are the CTLA-4 and the PD-1/PD-L1 (10).

Cetuximab is a monoclonal antibody that blocks the EGFR and is used in the treatment of metastasis, together with radiotherapy and chemotherapy. Another immunotherapy option includes the use of check-point inhibitors, which block PD-1 and CTLA-4 receptors from activating T cells. The PD-1 molecule is expressed in various immune system cells, particularly in cytotoxic T cells, and interacts with PD-L1 ligands, expressed by tumor cells, and with PD-L2, expressed by macrophages and dendritic cells. The interaction of PD-1 with PD-L1 and PD-L2 reduces the T-cell activity and promotes immune tolerance to the tumor. Therefore, if the PD-1 inhibitor function is inhibited, the T-cell response against the tumor can be achieved (11).

Nivolumab and pembrolizumab are associated with platinum and are anti-PD-1 antibodies. They are used as check-point inhibitors in the treatment of relapsed and metastatic HNSCC. They respond better in patients with positive PD-L1 tumors, even in the presence of HPV (9).

## Mechanisms of tumor escape and invasion

HNSCC has a high capacity of cell infiltration from the immune system. The tumor epitopes created by DNA alterations, some of which originate from viruses, induce the formation of new proteins that act as antigens whose tolerance is incomplete, as well as peptides not present in the human genome (10).

If an anomalous mutation occurs, the epithelial cell goes into apoptosis, is recognized by the immunosurveillance mechanism as an anomaly, and undergoes phagocytosis. If the immune system fails to make this recognition, the altered cell continues to develop and multiply itself, which can result in HNSCC (11,12). The immunoedition of cancer takes place by elimination, balance, and escape. In the elimination stage, the tumor grows invasively and requires a greater blood supply, which is provided by peritumoral vasculogenesis induced by the cancer itself. In the escape stage, the tumor can no longer be controlled by the immune system and spreads throughout the organism (13).

The T lymphocytes and NK cells recognize the cancer cells and produce interferon gamma (IFNγ) and cytokines. NK cells, macrophages, dendritic cells, CD8+ T lymphocytes, and other effector cells of the immune system migrate to the tumor region and cause its necrosis. The cytokines, primarily IFNγ, contain angiostatic properties and block the formation of blood vessels around the tumor, leading to the death of tumor cells. The local dendritic cells cause the phagocytosis of tumor cells and migrate to regional lymph nodes when antigens appear. Tumor necrosis takes place by inducing the death of tumor cells through cytolytic action capable of destroying cells with strange peptides on the surface, through two main mechanisms: facilitating the entrance of ions and water in the cell and stimulating cell apoptosis. The activated TCD8+ lymphocyte produces two proteins involved in this process, perforin and granzyme, which are concentrated in cytoplasmic granules linked to the cell membrane through exocytosis, and causes cell lysis. The destroyed tumor cells and their fragments are removed by the dendritic cells and are presented to the CD4+ and CD8+ specific antigen T lymphocytes (13). In the lymph nodes, the dendritic cells induce the differentiation of CD4+ T lymphocytes in TH1, which positively regulates the CD8+ T cells (13,14).

The HNSCC cells inhibit the immune system by means of innate and adaptive pro-tumor cells (14). The HNSCC microenvironment contains suppressor myeloid stromal cells, immature dendritic cells, and T lymphocytes associated with the tumor. These immunological cells play an important role in disease outcome. Depending on the activation of these cells and the surrounding signaling, the effect can be favorable or unfavorable to tumor progression and metastasis (15).

The cytotoxicity of NK cells is reduced by the decreased expression of class 1 MHC induced by tumor cells. Dendritic cells have a critical function, but in HNSCC, this function is hindered. Macrophages associated with the tumor produce IL-10 and secrete extracellular matrix proteases and serine protease associated with the most advanced degrees of tumor and metastasis (16). CD8+ T cells have no tumoricidal activity, due to the tumor's immunosuppressing mechanism, by means of PD-1 receptor, which links to PD-L1 ligand expressed by tumor cells (17). HNSCC is associated with few lymphocytes in peripheral blood, but memory T cells are highly present, suggesting the recognition of T cells (3).

## Strategies of immunostimulation

The recognition of immunosuppression in the initial phase of HNSCC allows for strategies of immunostimulation and tumor treatment (18). The PD-1 proteins transmembrane ligands, expressed by T-lymphocytes, inhibit the immune response when they join with their PD-L1 and PD-L2 ligands, expressed predominately by tumor cells, diminishing the defense mechanisms of the immune system due to the loss of cytotoxic activity of T lymphocytes. These mediators are part of the tumor microenvironment and can be expressed by both tumor cells and immune system cells (19). The expression of these proteins is greater in HNSCC than in peripheral blood, being directly proportional to tumor stage. The association between HPV and PD-L1 was observed in oropharynx tumors and is a prognostic factor for worse outcome (20). Therefore, PD-L1 expression is a predictive independent marker of tumor evolution, whose distribution varies between 46 and 87% (21).

This protein PD-L1 is expressed not only by all HNSCC cells but also by macrophages and T lymphocytes in this tumor (22). It is relevant to characterize the activation of T lymphocytes that express PD-1 in the activated or inhibited PD-L1 receptor and how PD-1 interacts with PD-L1 and PD-L2 (23).

## Clinical trials with anti-PD1 immunotherapy

In the CheckMate 141 trial, stage III, a better clinical response to treatment was observed with the anti-PD-1 immunotherapeutic drug nivolumb, used as a monotherapy (3 mg/kg every 2 weeks). However, chemotherapy using platinum salt proved more effective in patients that expressed higher levels of PD-L1. In the KEYNOTE 12 study, involving a cohort of 132 patients with recurrence or metastasis treated with the anti-PD-1 immunotherapeutic drug pembrolizumab, 200 mg every three weeks, a greater survival was observed in patients that expressed PD-L1 in the tumor microenvironment. However, results of clinical trials related to the predictive value of PD-L1 are very heterogeneous. Another limitation is the multiplicity of anti-PD-L1 antibodies and the lack of therapeutic standardization. In a cohort of HNSCC patients treated with the anti-PD-1 immunotherapeutic drug pembrolizumab, the simultaneous expression of PD-L1 and PD-L2 were directly correlated with patient survival and improvement in the immunotherapeutic response (24).

Based on outcomes from trials with pembrolizumab and nivolumab, the Food and Drug Administration (USA) approved these immunotherapeutic drugs for the treatment of the metastatic recurrent forms of the disease. Their efficacy is limited to a subgroup of patients and to biomarkers definition. Tumors with PD-L1 expression have a greater probability of responding to inhibitors of the PD-1/PD-L1/PD-L2 axis, although the absence of PD-L1 expression is not an indicator of resistance to PD-1 inhibitors (25).

## Biomarkers of immunotherapy response

The inflammatory phenotype of T cells is proposed to be a response biomarker for immunotherapy (26,27). Studies conducted in melanoma suggest that therapy is beneficial in patients with pre-existing anti-tumor T-cell response, as shown by basal CD8+ T cell infiltration in the tumor microenvironment (28-30). A prospective study of the tumor microenvironment in HNSCC patients described CD8+ T-cell tumor infiltration in patients expressing the PD-1 molecule. These patients had a greater survival compared to those without infiltration, regardless of the triggering factors, such as HPV, smoking, and alcoholism (31,32).

IFNγ is a cytokine produced by activated T cells and NK cells in the tumor microenvironment and plays an important role in anti-tumor immunity. IFNγ leads to an increase in the expression of PD-L1/2 in the tumor and in the tumor infiltrate. Immune resistance takes place after the migration of T cells to the tumor environment, indicating an immune response regulation effect (33-35). These findings, together with results in patients with melanoma, suggest that patients with a more exacerbated inflammatory response achieve a better therapeutic response to PD-1 blockers (5,36).

HNSCC cells can lead to aberrant hematopoiesis, which inhibits the immune system due to the recruitment of pro-tumor cells (37,38). In the peripheral blood of HNSCC patients, not only are T cells detected in lower concentrations, but also a high percentage of memory effector T cells are observed. When patients have a lower inflammatory process, the immune resistance appears to be attributed to a lower migration of effector T cells (37,38).

## Final considerations

Immunotherapy is recommended for patients with locally advanced and metastatic HNSCC and has been evolving with T lymphocyte PD-1 inhibitors, the recommendation of which depends on reliable biomarkers that must be identified in order to achieve good therapeutic results.
